# Neuroimaging of decoding and language comprehension in young very low birth weight (VLBW) adolescents: Indications for compensatory mechanisms

**DOI:** 10.1371/journal.pone.0185571

**Published:** 2017-10-02

**Authors:** Helene van Ettinger-Veenstra, Carin Widén, Maria Engström, Thomas Karlsson, Ingemar Leijon, Nina Nelson

**Affiliations:** 1 Department of Medical and Health Sciences, Linköping University, Linköping, Sweden; 2 Center for Medical Image Science and Visualization (CMIV), Linköping University, Linköping, Sweden; 3 Department of Clinical and Experimental Medicine, Pediatrics, Linköping University, Linköping, Sweden; 4 Department of Behavioral Science and Learning, and Linnaeus Centre HEAD, Linköping University, Linköping, Sweden; 5 Department of Quality and Patient Safety, Karolinska University Hospital, Stockholm, Sweden; University Children's Hospital Tuebingen, GERMANY

## Abstract

In preterm children with very low birth weight (VLBW ≤ 1500 g), reading problems are often observed. Reading comprehension is dependent on word decoding and language comprehension. We investigated neural activation–within brain regions important for reading–related to components of reading comprehension in young VLBW adolescents in direct comparison to normal birth weight (NBW) term-born peers, with the use of functional magnetic resonance imaging (fMRI). We hypothesized that the decoding mechanisms will be affected by VLBW, and expect to see increased neural activity for VLBW which may be modulated by task performance and cognitive ability. The study investigated 13 (11 included in fMRI) young adolescents (ages 12 to 14 years) born preterm with VLBW and in 13 NBW controls (ages 12–14 years) for performance on the Block Design and Vocabulary subtests of the Wechsler Intelligence Scale for Children; and for semantic, orthographic, and phonological processing during an fMRI paradigm. The VLBW group showed increased phonological activation in left inferior frontal gyrus, decreased orthographic activation in right supramarginal gyrus, and decreased semantic activation in left inferior frontal gyrus. Block Design was related to altered right-hemispheric activation, and VLBW showed lower WISC Block Design scores. Left angular gyrus showed activation increase specific for VLBW with high accuracy on the semantic test. Young VLBW adolescents showed no accuracy and reaction time performance differences on our fMRI language tasks, but they did exhibit altered neural activation during these tasks. This altered activation for VLBW was observed as increased activation during phonological decoding, and as mainly decreased activation during orthographic and semantic processing. Correlations of neural activation with accuracy on the semantic fMRI task and with decreased WISC Block Design performance were specific for the VLBW group. Together, results suggest compensatory mechanisms by recruiting additional brain regions upon altered neural development of decoding for VLBW.

## Introduction

Very low birth weight (VLBW <1500 g) children are at significant risk of cognitive and behavioral impairments such as language processing and reading deficits which persist into adolescence. They are also more likely to require special assistance in school [1–5]. Language processing is essential for reading, learning and school performance [6], and language function in preterm-born individuals is suggested to develop differently compared with term-born children. However, VLBW (often caused by preterm birth, a slow prenatal growth rate, or a combination thereof) in combination with preterm birth does not affect all types of language ability negatively. We investigated VLBW in preterm born children in Sweden, where around 6% of all births are preterm (*i*.*e*. <37 weeks’ gestational age), and 0.6% are born with a very low birth weight of less than 1500 g. The neonatal mortality (within 27 days postpartum) rates for VLBW neonates was 28% in 2014 [7]. The impact of preterm birth on language and reading ability in surviving neonates has been reviewed by Lee and colleagues [8]. They identified inconclusive results from studies on language and reading ability, and where thus motivated to conduct an extensive study on various cognitive and language functions, on a group of 65 adolescents born preterm with low birth weight (<36 weeks of gestation, <2500 g, mean age = 12.2) in comparison to 35 term-born control adolescents (mean age = 12.6). The authors found that reading comprehension, different language skills, linguistic processing speed, performance IQ (which includes the Wechsler Intelligence Scale of Children (WISC) Block Design test), and verbal IQ (which includes the WISC Vocabulary test) were significantly impaired in the preterm group, but receptive vocabulary was not. Moreover, the study showed that linguistic processing speed, verbal memory, and reading comprehension varied with degree of prematurity, however receptive vocabulary, syntactic comprehension, and decoding did not show dependence on degree of prematurity. The degree of prematurity variance effect was independent for sex, maternal education, and IQ. Another longitudinal study investigated a large cohort of 322 very preterm VLBW children at age 8, 12, and 16 and compared them to 41 term-born controls on cognitive and language tests [9]. Receptive vocabulary did improve significantly over time, and a catch-up was observed for cognitive tests for the VLBW children that were protected by high maternal education, non-minority ethnicity, and low neurosensory impairment. Regarding reading abilities; the authors showed that phonological awareness phonemic decoding was impaired, but not sight word reading. In Swedish samples, IQ, Performance IQ, and Vocabulary are shown to have a correlation with VLBW reading performance [10,11].

Several study outcomes support the hypothesis that VLBW and preterm born children are able to catch up with their term-born peers regarding certain impaired language abilities. Improvement of cognitive functions in preterm children was studied by Ment and colleagues, who showed in their longitudinal study on preterm VLBW followed up to 8 years after birth that most of these children improved on their verbal and IQ performance scores over the years; only children who experienced severe brain injury did not [12]. Samuelsson and colleagues investigated whether VLBW children caught up with their peers regarding reading ability, by comparing 56 VLBW and 52 normal birth weight children at age 9 and 15 [10]. Indeed, most between-group reading ability differences observed at age 9 were no longer significant at age 15; there was an improvement of both reading comprehension and word-recognition found for the VLBW group when controlled for individual IQ differences.

For a basic understanding of reading comprehension, we refer to the *simple view of reading*, which presents a simple, yet powerful model where reading comprehension is the product of decoding skills (single word reading) and language comprehension (sentence/discourse interpretation) [13]. Even though the development of reading is likely more complex than this model can describe [14], the *simple view of reading* has proven useful to classify reading disability [15]. Successful decoding of a word is a combination of phonological (related to meaningful sounds) and orthographic (related to word form) information being processed correctly [16]. These two pathways appear to exist separately of each other according to the dual route model [17], however their functioning tends to be interdependent [18]. The phonological pathway is important during initial stages of reading. When all word forms are unfamiliar, the word is sounded out in fragments, piece-by-piece. Later, when word forms are acquired, the orthographic pathway enables fluent reading of familiar words by automatically retrieving or mapping meaningful strings of letters [18]. Underdevelopment of either pathway can lead to reading ability problems such as developmental dyslexia [19]. Cartwright extended on the *simple view of reading* by showing that the flexibility of an individual to simultaneously process phonological and semantic features of a word contributes to better reading comprehension [20].

The neural division of the dual decoding model into a dorsal and ventral system is relatable to *the simple view of reading* and other current models of reading [21]. The slow and effortful dorsal (temporoparietal) system can process phonological information and map this onto the semantic structure. The fast responding ventral (occipitotemporal) system is responsive to orthographic features and takes over when reading becomes automatic [22]. A recent review supports this neural dual decoding route and attributes phonological processing to the perisylvian angular and supramarginal gyrus, and to the temporal gyri; orthographic processing to occipital regions including the fusiform gyrus; and semantic processing also to perisylvian regions and inferior frontal gyrus [21]. Moreover, lower neural efficiency of beginning readers was characterized by the recruitment of more brain regions during decoding stages.

In children born preterm there is evidence for impaired decoding and phonological processing [11]. As discussed before, some of these impairments may be overcome or circumvented when children approach adulthood [10]. Decoding and phonological processing can advantageously be studied by functional magnetic resonance imaging (fMRI). However, to this date there is only a small set of fMRI studies that have investigated the neural correlates to phonological and semantic processing in children or adolescents born preterm and in specific with VLBW. Two of these VLBW studies investigated phonological and semantic processing in preterm born children with the use of an auditory story listening task, with a semantic condition (intelligible stories) and a phonological condition (unintelligible stories due to reordering of all phonemes) [23, 24]. The first auditory listening study found an indication for an activation/deactivation pattern during the semantic task in the group of 26 VLBW preterm born children (~ 8 years old) that resembled the activation/deactivation pattern of the 13 participants in the matched control group during phonological but not semantic processing [23]. The authors hypothesized that the preterm born children used phonological processing pathways during semantic processing. This pattern was observed mainly in frontal regions–however not in regions primarily attributed to reading–and showed to be independent of maternal education or intraventricular hemorrhaging treatment. A second study from the same research group on a different group of 12-year old preterm VLBW adolescents (n = 11) found comparable results, but now these findings were also located in regions that are associated with reading [24]. This study showed that when the preterm born children engaged in semantic processing, they showed less activation than term-born controls. During phonological processing, preterm born children showed more activation than term-born controls, in both regions underlying semantic as regions involved in phonological processing. Moreover, VLBW compared to controls showed activation of the bilateral temporal gyri but not the frontal cortex during phonological processing, whereas the same temporal regions were activated in controls but not in VLBW during semantic processing. The authors argue that by recruiting semantic processing regions in bilateral temporal gyri, the preterm group may compensate for poor phonological processing [24]. An fMRI investigation of visual rather than auditory processing in 18-year olds performed a direct comparison of neural activation of six VLBW preterm born adolescents to six term-born controls [25]. The VLBW adolescents born preterm showed additional activation in right-hemispheric non-reading-specific regions during phonological and orthographic processing of visual stimuli compared to term-born controls. Furthermore, the preterm group showed reduced involvement of a part of the left fusiform gyrus (the visual word form area). These fMRI studies highlight compensatory mechanisms as an explanation for atypical neural activity in preterm brains, and imply an underdeveloped or modulated functionality of the phonological and semantic systems. This is hypothesized to be a transposed activation pattern in VLBW born children in frontal and temporal regions and resulting in poor phonological processing skills [23–25]. Functional connectivity studies show overall increased patterns of connectivity for preterm adolescents, in particular connectivity with right-hemispheric regions. The lateralization of connectivity was repeatedly shown to correlate with language abilities [26]. Moreover, lateralization is likely altered in very preterm born children, indicating the need of bilateral investigations [27]. However, the contribution of visual decoding processes in VLBW in relation to neural activation remains to be investigated.

Reading ability is essential for school performance and for a normally functioning daily life. Therefore, there is a clear need of extending the findings of aberrant phonological processing in preterms born with VLBW to a between-group study investigating salient aspects of reading such as the current study provides. We expect that children born preterm with VLBW experience delayed or impaired development of reading abilities, for which they have not yet fully learned to compensate during young adolescence [9,10,12]. We wish to understand whether visual decoding and comprehension aspects of reading are represented differently in the brain of young VLBW adolescents who just completed elementary school, compared to normal birth weight controls. We expect that the phonological processing pathways are affected by preterm birth with VLBW, and if this is a persistent disruption of the pathway, this would have an impact on both phonological and orthographic decoding skills of young adolescents with VLBW, since orthographic decoding in beginning readers is developed through phonological decoding of new words.

### Hypotheses

We hypothesize that both phonological and orthographic decoding abilities are affected by VLBW in young adolescents born preterm and this group will therefore exhibit lower performance on the decoding tasks. However, if compensation processes have started, we expect that young VLBW adolescents compensate for a dysfunctional phonological processing system by recruiting additional brain regions during phonological and semantic processing [21,23–25]. Lastly, we expect these young VLBW adolescents to show evidence of cognitive problems typical for individuals born preterm on the conventional WISC by achieving lower scores, especially on the Block Design subtest, in concordance with previous results from our VLBW group [11]. A difference in cognitive functioning between groups may be related to separate neural differences in aspects of reading ability, as reading ability is related to cognitive abilities.

In the present study, our aim was to examine neural correlates to components of reading ability, namely decoding and language comprehension in young adolescents with VLBW and relate VLBW and reading ability to other cognitive measures.

## Materials and methods

This study is part of a longitudinal follow up of a cohort of VLBW (<1500g) infants born 1998–1999 in the southeast region of Sweden [11]. This region has five hospitals with obstetric and pediatric departments, including one level-three university hospital with a regional intensive care unit. The study protocol was approved by the Regional Ethical Review Board of Linköping, Sweden (Registration number 2011–3731), and oral and written informed consent was obtained from the participants and one of their parents. The families were informed that they could withdraw from the study at any time.

### Participants

Perinatal data for both groups were collected from medical registers following parental approval, and reported together with demographics in Table 1. All participants had to be fluent in Swedish. Exclusion criteria included reported concomitant neurological or psychiatric illness, and metal implants that could interfere with the fMRI investigation. All children attended regular schools.

**Table 1:** Perinatal and performance data for the included participants and the original cohort.

Variables	VLBW cohort	VLBW current study	NBW cohort	NBW current study
n	50	13	51	13
Girls, n (%)	32 (64%)	7 (54%)	32 (63%)	8 (62%)
Gestational age, weeks + days (SD, days)	29+0 (15)	29+2 (15)	40+1 (9)	39+6 (9)
Birth weight, g (SD)	1079 (289)	1046 (347)	3547 (416)	3559 (506)
Small for gestational age, n (%)	27 (54%)	10 (77%)	0	0
Extra low birth weight < 1000 g, n (%)	18 (36%)	6 (46%)	0	0
Prenatal dexamethasone, n (%)	32 (78%)^†^^9^	9 (69%)^†^^1^	0	0
Respiratory distress syndrome, n (%)	25 (50%)	6 (46%)	0	0
Surfactant use, n (%)	16 (32%)	2 (17%)	0	0
Treated patent ductus arteriosus, n (%)	9 (18%)	0	0	0
Bronchopulmonary dysplasia, n (%)	16 (32%)	4 (31%)	0	0
Septicemia, n (%)	16 (32%)	4 (31%)	0	0
Necrotizing enterocolitis, n (%)	0	0	0	0
ROP > grade 1, n (%)	4 (8%)^†^^2^	0	0	0
Intracranial bleeding, n	8 (16%)^†^^5^	2 (15%)^†^^2^	0	0
Intracranial bleeding grade 1, n	7 (14%)	2 (17%)	0	0
Intracranial bleeding grade 2, n	0	0	0	0
Intracranial bleeding grade 3, n	1 (2%)	0	0	0
Periventricular leukomalacia, n (%)	1 (2%)^†^^6^	0^†^^2^	0	0
Maternal education: Elementary, n (%)	2 (15%)	10 (20%)	0	2 (4%)
Maternal education: Gymnasium, n (%)	5 (39%)	23 (46%)	5 (38%)	21 (41%)
Maternal education: University, n (%)	6 (46%)	17 (34%)	8 (62%)	28 (55%)
WISC a: Block Design Raw Scores, correct answers mean ± SD	28.0^b^ ± 12.1	33.7^c^ ± 12.8	45.2^b^ ±6.9	48.8^c^ ± 7.0
WISC a: Vocabulary Raw Scores, correct answers mean ± SD	24.0^b^ ± 7.7	35.7^c^ ± 12.5	29.9^b^ ± 4.0	43.9^c^ ± 10.5

†n) Data missing for n participants.

a) Wechsler Intelligence Scale for Children, for version see annotation per column.

b) At age 9: Wechsler Intelligence Scale for Children, WISC-III. (3rd ed.) Swedish manual. Stockholm: Psykologiförlaget, 2002.

c) at age 13WISC–IV (4th ed.). Swedish manual. Stockholm: Pearson Assessment and Information AB, 2007. VLBW = very low birth weight (<1500 g), NBW = normal birth weight, n = number, SD = standard deviation, g = gram, ROP = retinopathy of prematurity.

#### VLBW group

During the intake period (1998–1999), 103 VLBW infants were born in the southeast region of Sweden, of which 93 (90%) survived the neonatal period. At the age of both 7 and 9 years, this cohort was enrolled in prior studies focusing on behavior, cortisol levels, cognition, and reading comprehension; 50 children completed the follow-up at both 7 and 9 years of age [11,28]. Our current follow-up study of children age 12–14 –who are now participating as young adolescents–includes 13 of the 50 children studied at ages 7 and 9; 25 declined, 11 did not respond, and one withdrew. The mean age of the 13 included VLBW was 13.5 years (SD = 0.6). Cognitive assessments were obtained of all 13 participants, fMRI data were collected from 11, and brain volume data from 12 participants. All VLBW participants were right-handed, exclusion from the fMRI analysis was due to excessive head movement or technical problems. The relative high rate of VLBW adolescents that were small for gestational age has been observed in the larger cohort tested at age 7 [11]. Since that cohort showed reading impairments, SGA was tested for correlation with reading variables and no significant correlations were found in the study on 7-year olds [11]. Our current study sample size prohibited further investigation of SGA in this study. We confirmed that our VLBW group was representative for the initial cohort tested at age 7, by performing a statistical comparison on all perinatal variables that were available to us in this study. We performed t-tests on birth weight and gestational age, Mann-Whitney U-tests on ordinal factors of surfactant use, septicemia, and maternal education, and Chi-square tests on SGA, extra low birth weight, respiratory distress syndrome, bronchopulmonary dysplasia, retinopathy of prematurity, intracranial bleeding (collapsed grading), and periventricular leukomalacia. None of these tests showed significant differences in relation to the inclusion factor.

#### Normal birth weight group

The former studies of children age 7 to 9 included a control group of 51 normal birth weight (NBW, ≥ 2500 g) children, which was selected as follows: for each VLBW participant, a child living in the same municipality was selected from the national birth register for comparison. These NBW children, who were matched for sex and number of siblings, were selected on the basis of being born as close in time to the VLBW child as possible and with no diagnosis in the standard maternity protocol. Of this group, 13 NBW agreed to participate in this current follow-up at age 12–14; seven had declined during earlier studies, 21 declined for the present study, nine did not respond and one child had moved abroad at the time of recruitment and was excluded. The mean age of the included group of 13 NBW was 13.0 years (SD = 0.2). Cognitive assessments and MRI data from all 13 NBW adolescents were included in the analyses, and data from 12 NBW participants were included in the brain volume analysis, one participant having been excluded due to technical problems. One NBW participant was left-handed, however since this individual showed no indication of right-lateralized activation on the fMRI tasks, we found no justification to exclude this participant from the study.

### Functional imaging

#### FMRI Tasks

Based on previous observed reading problems [11], a language task based on a Swedish test to screen for dyslexia was developed for this study [29]. This test included a word pair task and a recognition task (the latter will be presented elsewhere). The word pair task comprised three language conditions, namely a phonological choice condition, an orthographic choice condition, a semantic judgment condition, and a line orientation baseline condition. The phonological and orthographic conditions were similar to the tasks that previously have been used to characterize reading problems in VLBW [10,11]. The tasks were presented through rear projection on a screen behind the head of the participants. The screen was visible to the participants through a mirror mounted on the head coil.

**Fig 1:**
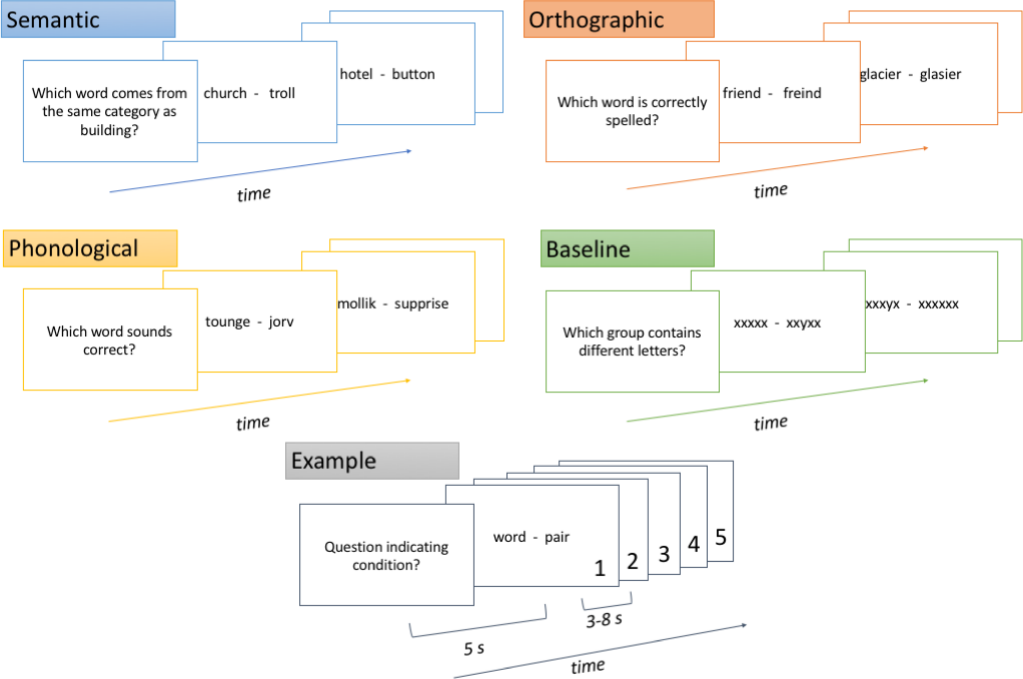
Schematic overview of the word pair task used during the fMRI session. The different blocks for each language choice condition are shown, the example block shows the timing in seconds for the blocks, and the sequence of 5 word pairs after each question.

The word pair task consisting of the described four language choice conditions was presented according to a block design (Fig 1). Each condition block started with a specific question presented to the participants for five seconds. Next, a word pair was shown; one of the words was the correct answer to the question, all real words were proper nouns. The participants answered by pressing one out of two buttons on a response box (Lumina LU444-RH, Cedrus Corporation, San Pedro, U.S.A.) with their index finger (for the word presented at the left of the screen) or their middle finger (for the word presented at the right) of their right hand. The mean duration of word pair presentation was five seconds, but varied from three to eight seconds according to a Poisson distribution. There were five word pairs per block, all relating to the initial question. The semantic condition asked for word categories, each semantic block asked about a different category. The orthographic condition investigated spelling with a word pair of a correctly and an incorrectly spelled word. The phonological condition asked "Which word sounds correct?” after which pairs of pseudo-words were presented. One of the pseudo-words was a real word when sounded out, while the other was not. The line orientation condition served as a baseline condition and showed two strings of "x", in one string one "x" was replaced by an "y".

Each new stimulus was separated from the previous by a short 100 ms fixation break, while a five second pause separated the condition blocks from each other. After presenting four condition blocks (semantic, orthographic, phonological, and baseline), there was a 10 second break, followed by a new set of the four condition blocks presented in a different order and with different words (and a different category for the semantic block). In all, four condition blocks were presented four times, resulting in a total of 20 pairs of words for each condition, to make a grand total of 80 presented word pairs.

To become familiar with the task, all participants completed an off-line training session prior to the fMRI session. The training session words were different from those presented during the scanning procedure.

#### Image acquisition

All examinations were conducted at the Center for Medical Image Science and Visualization (CMIV, Linköping University), located at Linköping University Hospital, Sweden. Images were acquired using a Philips Ingenia 3.0 Tesla scanner with a standard head coil, which was halfway the study replaced by a head-neck coil due to technical complications, this affected both the VLBW and the NBW group equally. Structural and functional brain images were obtained during a scanning session of approximately 40 minutes.

The functional images were compiled using a single-shot gradient-echo echo planar image (EPI) sequence sensitized to T_2_* blood oxygen level dependent (BOLD) signal changes. Whole brain coverage was obtained with 34 or 35 slices with a 0.3 mm slice gap (10% voxel size). Repetition time = 2 s, time to echo = 35 ms, flip angle = 75°, voxel size = 3x3x3 mm^3^, acquisition matrix = 72x68 voxels, number of dynamics = 248. The slices were aligned between the floor of the sella turcica and the posterior angle of the fourth ventricle. The structural images were collected with T1-weighted sequences covering the whole brain with the following parameters: repetition time = 7.5 ms, time to echo = 3.5 ms, flip angle = 8°, voxel size = 1.1x1.1x0.6 mm^3^, acquisition matrix 228x227 voxels, with 301 slices obtained.

#### Preprocessing

Preprocessing and statistical analysis were performed in SPM8 (www.fil.ion.ucl.ac.uk/spm/software/spm8). The DARTEL toolbox was used for preprocessing [30], with default settings for EPI data as outlined in the SPM8 manual (www.fil.ion.ucl.ac.uk/spm/doc/spm8_manual.pdf). We used DARTEL to create a template of extracted grey and white matter from the anatomical images of all participants in this study (both VLBW and control group) with the default number of 6 outer iterations. The template was then normalized within the DARTEL toolbox to a 2x2x2 mm^3^ Montreal Neurological Institute (MNI) template and smoothed with an 8 mm full width half maximum (FWHM) Gaussian kernel. In SPM, the individual realigned and resliced EPI data from the fMRI tasks were coregistered and then normalized to 2x2x2 mm^3^ MNI space with help of the normalized DARTEL template and smoothed with an 8 mm FWHM Gaussian kernel.

### Cognitive assessments

Components of intellectual capacity was assessed on the same day prior to the fMRI session, using Vocabulary and Block Design subtests from the Wechsler Intelligence Scale of Children (WISC-IV, Wechsler 2003). WISC Vocabulary asks for word definitions, and measures verbal comprehension. WISC Block Design measures visuo-spatial processing by asking to recreate patterns with colored blocks. Furthermore, the parents of each participant were asked to fill in a questionnaire regarding their child’s health status, family constellation and school situation. The participant data, cognitive measures from the WISC tests, and fMRI performance data can be found in S1 Table.

### Statistics

**Fig 2:**
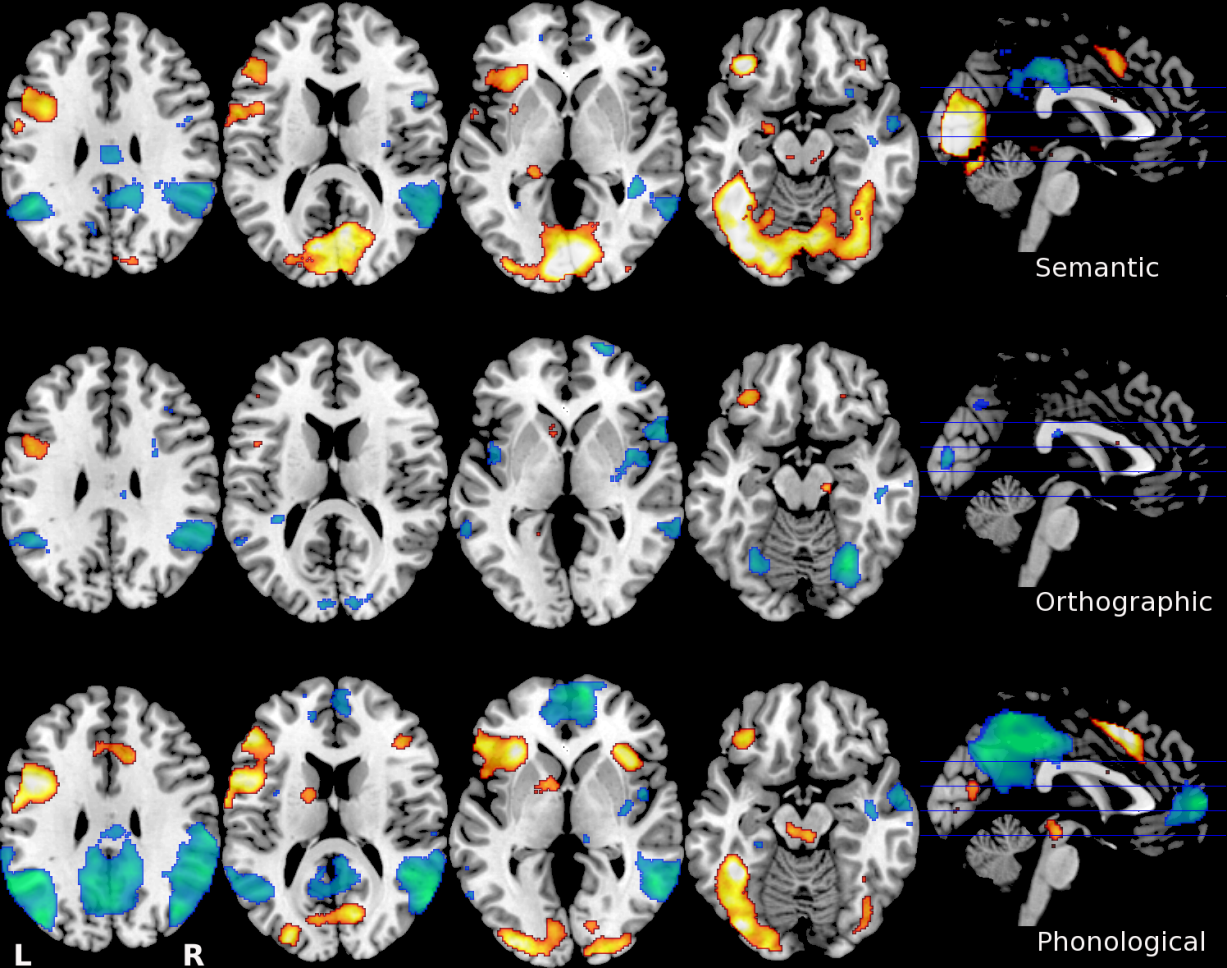
Neural activation (warm/orange) and deactivation (cool/blue) during the three fMRI language tasks (semantic, orthographic, and phonological processing) for all participants. The locations of the transversal sections are shown in the sagittal midline section on the right. Locations of transversal section are identical for all tasks. For visualization purposes to show the extent of the activation, these images are thresholded at p = 0.001 uncorrected. L = left hemisphere, R = right hemisphere.

The individual preprocessed images were analyzed for brain activation related to sentence processing by applying contrasts between the language conditions and the line orientation baseline. This resulted in three contrast images per participant for semantic > baseline, orthographic > baseline, and phonological > baseline. Head movement parameters were filtered out by entering those into the analysis as multiple regressors of no interest. To qualify as an exclusion criterion, excessive head movement was defined as > 3 mm in-plane motion; since this interfered with successful normalization. Group activation for all participants per language condition in the whole brain at a significance threshold of 0.001 uncorrected was used for multiple regression (Fig 2). Performance on the fMRI tasks was measured in terms of accuracy and reaction time. Accuracy was defined as the hit rate on the answers, which was calculated by subtracting the number of incorrect from correct answers for each condition and each participant.

Between-group differences were investigated per language condition with three multiple regression analyses, each with the inclusion of the following covariates: age, sex, accuracy on fMRI tasks (per condition), WISC Vocabulary Raw Scores and WISC Block Design Raw Scores. Age and sex were included to control for deviation from the original matched groups in the first study (at age 7), as this study was not aimed towards investigating age effects or sex differences. Accuracy on fMRI tasks, WISC Vocabulary Raw Scores and WISC Block Design Raw Scores are considered to be potential correlates to language performance, and therefore covariates of interest. All covariates were centered around the overall mean of that covariate. We investigated group and performance effects in 12 pre-defined regions of interest, six in each hemisphere, that we hypothesized to be most likely to show main effects. This assumption was based on the involvement of these regions in reading [22,31] and previous results observed in studies on language functioning in preterm children and adolescents [24,25]. The pre-defined regions were based on the Talairach Daemon labels atlas in the WFU pickatlas [32,33]. The regions were as follows: Inferior Frontal Gyrus, Middle Temporal Gyrus, Superior Temporal Gyrus, Angular Gyrus, Supramarginal Gyrus and Fusiform Gyrus in the left and the right brain hemisphere respectively. F-tests were applied to investigate the main group effect, any confounding main effects of age or sex, and main effects of the covariates of interest; accuracy on fMRI tasks, WISC Vocabulary Raw Scores and Block Design Raw Scores. Post-hoc t-tests were calculated to investigate direction of activation that was significant under the F-tests in our regions of interest, and to investigate interactions between group and the covariates of interest. All between-group F- and t-tests were thresholded per pre-defined region of interest at a peak-level significance threshold of p < 0.05, FWE corrected, with a minimum cluster extent of 10 voxels. Only for the interaction effects non-corrected post-hoc t-tests per group were made with age and the effect of interest as covariate, to verify the direction of the interaction.

The remaining statistical analyses were carried out using IBM SPSS Statistics 22.0. To test the hypothesis that the VLBW group had lower performance on the fMRI tasks, in the form of lower accuracy and slower reaction times, we applied a multivariate ANOVA including either of these two variables on all four different conditions (baseline, semantic, orthographic, and phonological) and tested between-group differences. The hit rate data underlying the accuracy on fMRI tasks measurement showed large variance, which necessitated a transformation of the data. A transformation of 1/(1.1—hit rate) showed to be the most optimal in reducing variance. For the 2-independent samples tests comparing WISC Block Design Raw Scores and WISC Vocabulary Raw Scores between groups, we opted to use the non-parametric Mann-Whitney U-test, since the small sample sizes per group (minimum of 11) makes it difficult to meet the assumption of normal distribution, and a significance threshold of p < 0.05 (one-tailed) was applied. Maternal education was tested with the Mann-Whitney U-test as well, since the data was categorical (categories: Elementary education, Gymnasium, Higher education).

## Results

### FMRI performance results

Accuracy on the fMRI tasks was above chance-level (10 out of 20 correct) for all participants, confirming active engagement in the task. Mean and range of correct answers for the whole group was as follows: semantic: 19.85 (18–20), orthographic: 19.62 (14–20), phonological: 18.85 (13–20).

The multivariate ANOVA concerning accuracy or response time on the fMRI tasks resulted in no significant between-group differences for any of the task conditions. Wilks' Lambda test: accuracy F(3,22) = 1.54, p = 0.23; response time F(3,22) = 0.33, p = 0.8. Accuracy range VLBW semantic 0.75–1; orthographic 0.5–1; phonological 0.05–0.8; NBW semantic 0.9–1; orthographic 0.7–1; phonological 0.3–0.95. However, there was a between-condition main effect both for accuracy F(3,22) = 17.13, p<0.001 and for response time F(3,22) = 214.42, p<0.001; which was significant at p<0.001 for post-hoc t-tests between every pair of conditions. Lower accuracy and longer reaction time was observed in ascending order for semantic, then orthographic, then phonological processing.

### Group activation

At an initial one-sample t-test investigating all participants, the semantic processing condition shows that activation occurred in the occipital lobe extending to the fusiform gyri, and in the left IFG. Deactivation clusters were observed in the inferior parietal lobule and in the posterior cingulate cortex. Orthographic processing activated the left IFG and clusters in the frontal lobe, and deactivated the right Brodmann area 9 in the frontal lobe and the inferior parietal lobule bilaterally. Phonological processing activated multiple clusters in the brain, including the anterior cingulate gyrus, the occipital lobe bilaterally, the left IFG and the left fusiform gyrus. Deactivation during phonological processing was observed in the posterior cingulate gyrus and bilaterally in the inferior parietal lobule.

**Fig 3:**
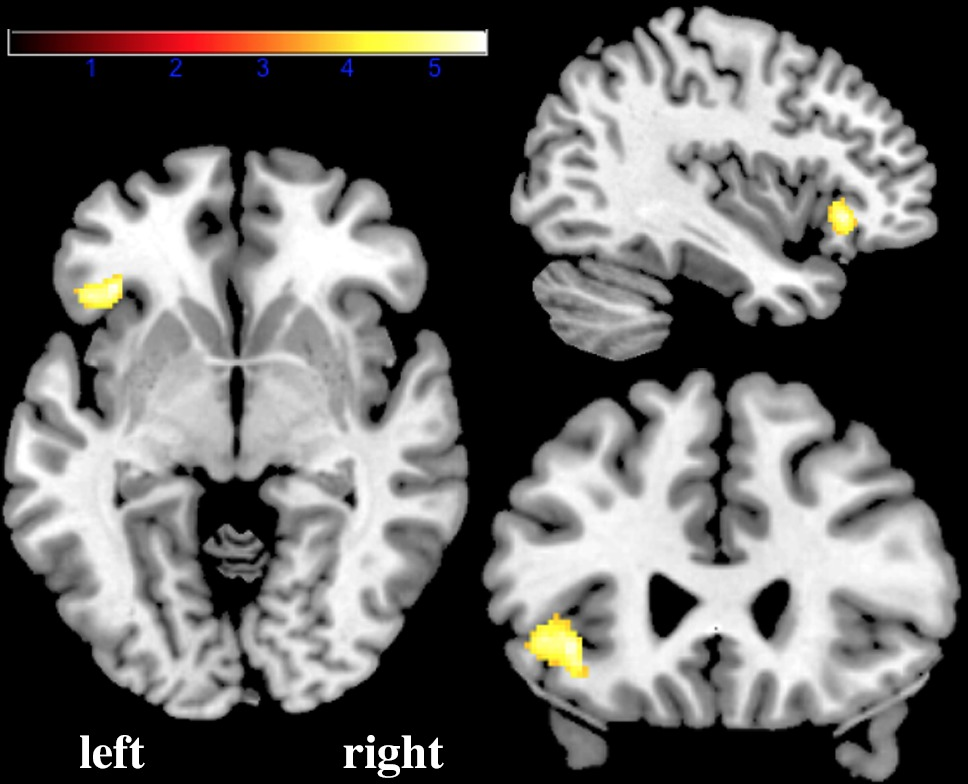
Activation in the left inferior frontal gyrus during phonological processing was greater for the very low birth weight group than for the normal birth weight group. A representative coronal, sagittal and transversal section at a region of interest analysis thresholded at p < 0.05 corrected for family-wise error rate.

**Fig 4:**
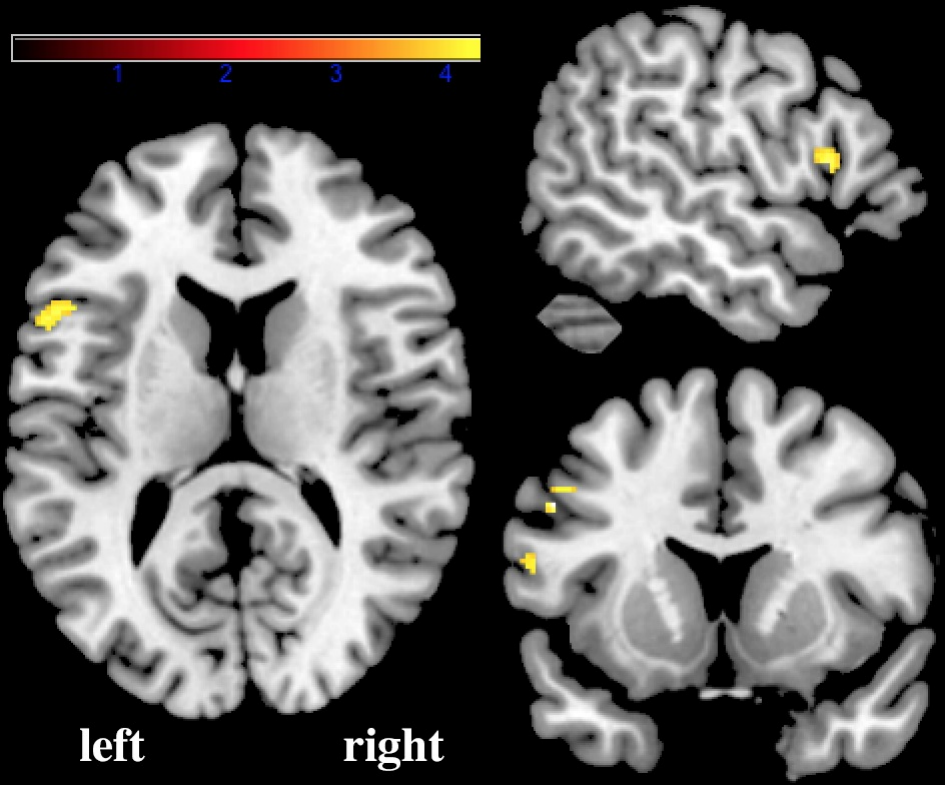
Activation in the left inferior frontal gyrus during semantic processing was greater for the normal birth weight group than for the very low birth weight group. A representative coronal, sagittal and transversal section at a region of interest analysis thresholded at p < 0.05 corrected for family-wise error rate.

**Fig 5:**
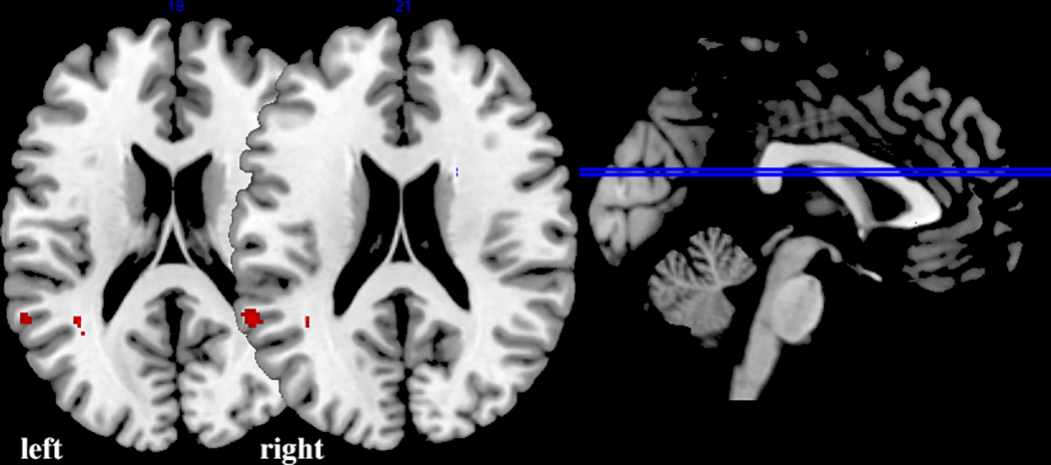
Main effect of Age during orthographic processing in left supramarginal and superior temporal gyrus. Representative transversal sections at a region of interest analysis thresholded at p < 0.05 corrected for family-wise error rate.

The multiple regression analyses compared between-group results per language condition, corrected for age and sex, and testing for performance effects. The VLBW group, compared to the control group, had increased phonological activation in the left IFG, decreased orthographic activation in right SMG, and decreased semantic activation in left IFG (see Figs 3 and 4 and Table 2 for clusters). Of the nuisance covariates age and sex, age did show a main effect only during orthographic processing, as increased activation in left SMG and left STG (Fig 5). No interaction of the nuisance variables with group was observed, nor was any of our reported effects of interest for orthographic processing in the left SMG or left STG.

**Table 2:** Region of interest analysis activated clusters for main and interaction related to reading.

Contrast	Region of interest	Size	peak Z	peak p (FWE)	MNI coordinates		
					x	y	z
**Group comparison VLBW > Controls**							
Phonological	Left IFG	301	5.53	0.036	-36	29	-8
			5.44	0.042	-45	26	-3
Orthographic	Right SMG	109	-6.54	0.002	47	-53	39
Semantic	Left IFG	40	-5.54	0.037	-50	12	33
**Nuisance variable Age**							
Orthographic	Left SMG	31	4.90	0.017	-60	-48	23
	Left STG	23	5.90	0.024	-38	-50	20
**Main effect covariates of interest**							
Phonological Block Design	Right SMG	14	-4.75	0.025	39	-53	30
			-4.46	0.039	36	-51	27
Orthographic Block Design	Right STG	56	5.91	0.023	50	12	-29
Semantic Accuracy	Left Angular	18	5.86	0.002	-35	-80	29
**Interaction effects covariates of interest with group**							
Orthographic Block Design	Right SMG	108	6.48	0.002	47	-53	39
Orthographic Vocabulary	Right SMG	110	6.54	0.002	47	-53	39
Orthographic Accuracy	Left SMG	11	-4.82	0.023	-38	-42	30
Semantic Accuracy	Left Angular	14	5.48	0.004	-34	-80	29

MNI = Montreal Neurological Institute, Size = Cluster size in voxels, FWE = Family-wise error corrected for multiple comparisons, VLBW = very low birth weight, IFG = Inferior Frontal Gyrus, SMG = Supramarginal Gyrus, STG = Superior Temporal Gyrus. Negative Z-values indicate an inversed effect. Covariates of interest are: WISC Block Design Raw Scores (Block Design), WISC Vocabulary Raw Scores (Vocabulary) and accuracy on fMRI tasks (Accuracy).

#### Performance effects and interaction with group

VLBW participants scored significantly lower than NBW on the WISC Block Design test (U = 31, p = 0.005). A main effect of performance on neural activation in regions involved in reading was observed for all three language conditions.

During phonological processing, lower performance on WISC Block Design was correlated to increased right SMG activation.

During orthographic processing, higher performance on WISC Block Design was related to increased activation in right STG. Also, there were interactions observed during the orthographic condition. There was an interaction effect observed of group x WISC Block Design performance as well as group x WISC Vocabulary performance in right SMG. Post-hoc testing revealed that this effect was not driven by a true interaction between group and WISC Block Design and WISC Vocabulary performance; apart from the group main effect in right SMG, there were also trends for increased right SMG activation related to lower WISC Block Design and WISC Vocabulary performance for both VLBW and control participants. An interaction during orthographic processing was observed for group x Accuracy on fMRI tasks in left SMG, more superior than the cluster related to age described above. Post-hoc testing revealed that there was a trend for left SMG activation related to lower accuracy for all participants, however per-group testing only showed this effect in the NBW group.

During semantic processing, higher accuracy during the semantic condition was related to increased activation in left angular gyrus, this region also showed an interaction of group x Accuracy on fMRI tasks, and post-hoc tests indicated that this effect was present mainly for the VLBW group.

## Discussion

The VLBW group showed increased activation in the left inferior frontal gyrus for the phonological condition, decreased activation in the right supramarginal gyrus for the orthographic condition, and decreased activation in the left inferior frontal gyrus for the semantic condition. A relation between WISC Block Design Raw Scores and altered right-hemispheric activation was found, also the VLBW group showed lower WISC Block Design Raw Scores than NBW. High accuracy on the semantic fMRI task in the VLBW group was characterized by increased activation observed in the left angular gyrus.

### Language task and cognitive performance differences

Our first hypothesis stated that we expected that VLBW is associated with decoding impairments, and if this impairment was still present during early adolescence, this would be visible in the VLBW group as decreased performance on the fMRI tasks (lower accuracy and slower reaction times), in specific during the phonological and orthographic decoding conditions. No evidence for decreased behavioral performance during any of the fMRI reading ability conditions was found, indicating that if there is a persisting decoding impairment as result of VLBW at the age of 12–14 years, the behavioral measures of our included language tests are insensitive to this effect.

Furthermore, we expected to find evidence of cognitive problems in the VLBW participants, and supporting this hypothesis was the finding of lower performance scores on the WISC Block Design test. This was also observed in a previous study that included WISC Block Design as a measure of Performance IQ in these individuals at age 7 [11]. Previous research has shown that there is a catch-up in several cognitive abilities for preterm children happening pre-adolescence [9,12], and we find no evidence for fMRI reading ability deficits related to low birth weight in preterm adolescents. In agreement with our neuroimaging findings discussed below of a relationship between WISC Block Design Raw Scores and aberrant neural activation, we hypothesize that decreased WISC Block Design performance is related to the development of changed functional activation patterns for reading ability compontents, however is not a correlate to reading ability performance. No between-group difference was found on the WISC Vocabulary test at age 12 unlike previous findings [10,11]. This is however likely to be influenced by our small group size.

### Functional neuroimaging results

Our study indicates a non-normal activation of regions in the IFG for VLBW individuals during different language tasks that is not translated as performance differences during this task. Therefore, the VLBW group may have a different functionality of the networks activated by the language tasks, that is not necessarily related to language problems. However, we found a correlation of higher accuracy on the semantic task with higher activation in the left angular gyrus during semantic processing. Therefore, we suggest that left angular activation during semantic processing is affected by semantic ability, this effect appears to be most pronounced in relation to VLBW and preterm birth.

The increased activation in the left IFG for phonological processing for VLBW is on the border between pars orbitalis and pars opercularis. The IFG encompasses both phonological and semantic processes, however according to the seminal review paper of Bookheimer [34], semantic functions are located more anterior and inferior, corresponding to the activation peak found for phonological processing in our study. These results support our hypothesis stating that VLBW adolescents recruit additional brain regions more than NBW do. However, this is not related to a difference in activation by the different language conditions, as both phonological and semantic processing activated the left inferior frontal gyrus. However, the increased activation in VLBW might be related to a need for computational back-up to aid a suboptimal functioning phonological decoding system. If this would be a compensatory strategy, it appears to be effective in this paradigm resulting in no performance differences between groups. Our findings showing recruitment of additional language-related regions during phonological but not semantic or orthographic processing by VLBW individuals, are consistent with previous indications of altered phonological processing networks in individuals born preterm [23–25]. It has also been hypothesized that high-performing individuals are characterized by more efficient brains [35,36]. This is exhibited as less neural activation in high performers during simple tasks; an activation pattern which is thought to preserve resources for more complicated tasks. This hypothesis may explain the finding of increased left IFG and left angular activation for VLBW, as a less efficient working brain for phonological and semantic processing, recruiting similar brain regions more intensely.

Seemingly an opposite effect; that of IFG activation decrease for VLBW during semantic processing, has not the same location as can be observed in Figs 3 and 4. These clusters of activation decreases are in pars opercularis and triangularis, which falls into the region that shows less specified results according to Bookheimer [34]. Pars triangularis is, like the rest of the IFG, activated during a myriad of language processes. These processes include phonological, syntactic, and semantic processing, and other processes such as mental imagery, and is in particular a pivotal area for language production and comprehension [34]. Pars triangularis is in specific hypothesized to be critical to phonologic and syntactic unification [37]. Activation in this region has previously been shown to be diminished in preterm born young adults, presumably reflecting a more effortful phonological approach needed by these individuals to complete the task [25]. These results provide some additional support for a future hypothesis suggesting a transposed phonological/semantic activation pattern of individuals born preterm, attributable to a functional switch towards activating semantic processing regions in the frontal or temporal lobe for phonological processing in VLBW individuals [23–25].

The decrease in activation observed for orthographic processing for the VLBW group was interestingly enough located in the right SMG. Left SMG activates during demanding semantic and syntactic tasks and is thought to initiate or process subvocalization in aid of language comprehension, however it is unclear if this function is strictly lateralized [31]. Bilateral SMG normally shows activation during phonological processing, in specific when making more demanding phonological decisions [38], but is also involved in nonlinguistic visuo-spatial processes [39]. In concordance with this latter function of the right SMG is our finding of an interaction effect of Group x WISC Block Design and Vocabulary performance in the same region. This interaction effect showed to be an increase of right SMG activation that increased with WISC Block design and WISC Vocabulary performance which was more pronounced for NBW controls. High performance on the WISC Block Design task means the presence of stronger visuo-spatial skills, and the use of these processes might be a strategy that NBW controls more than controls use to solve the orthographic choice task. We hypothesize that VLBW adolescents in contrast cannot use or process the orthographic input as visuo-spatial information, and therefore have to rely on other strategies for orthographic decoding. We hypothesize that VLBW adolescents have developed these alternative strategies during decoding development in their early childhood dependent on their Block Design and Vocabulary skills in concordance with previous findings [11,24].

The phonological task elicited the most widespread activation pattern in both groups, compared to the other language tasks. When we investigated the non-decoding semantic task, whole-group results showed that the semantic task activated mainly the occipital lobe extensively, despite not having deviating visual features (compared to other tasks or baseline). A partial explanation could be that the occipital activation originated from the adjacent fusiform gyrus, which is related to language functions such as word recognition. Since we applied a standard 8 mm Gaussian kernel smoothing appropriate for the voxel sizes in our data set, some smearing of the data over anatomical boundaries is to be expected.

### Limitations and strengths

For a between-group fMRI study, the group size is small. However, there is a dearth of fMRI research studies on the relation of reading ability and VLBW, and this study can contribute to an increased understanding of decoding and semantic processing. Of interest is the potential selection bias, which may have hindered participation of children with greater difficulties. In fact, our findings may have been amplified had children with more disabilities participated. A smaller study also limits the amount of testing that could be done while still maintaining power to reach significance. Consequently, we were unable to investigate the effect of perinatal factors on neural activation and performance, although we did observe that maternal education did not differ between groups. Since 77% of individuals in our VLBW group were small for gestational age, we were unable to determine whether our findings were related to preterm birth or specifically to intrauterine growth restriction, however the initial sample tested at age 7 also contained more than 50% individuals small for gestational age, which did not affect reading ability, cognition, or behavior [11]. The phonological processing task was challengingly difficult, evoking a more extensive activation pattern and spanning a broader range for hit rate. Neural development in VLBW in relation to decoding will need to be further investigated to test specifically for reading ability components. Nonetheless, we believe that the results that we observed in our study leads to an increased understanding of language-evoked neural patterns in VLBW brains, which may help to improve school interventions in order to achieve better academic and social achievements for children born preterm with learning disabilities.

Studies focusing on reading comprehension in VLBW children are needed to determine whether intervention during early school years–when semantic, orthographic, and phonological processing are still developing–can positively affect reading comprehension performance and accompanying neural functionality.

## Conclusions

Our study investigated how decoding and language comprehension aspects of reading ability were affected in young people born preterm with VLBW, and whether their neural pathways would reflect impaired decoding and language comprehension, in relation to cognitive performance. Since our language tasks did not show performance differences between groups for phonological decoding nor orthographic or semantic processing, if there were any decoding or reading ability deficits in children with VLBW (as this cohort of VLBW has shown reading and language impairments in the past) they might be compensated for at young adolescence. The observed increase of activation for phonological decoding is consistent with our hypothesis for an altered functionality of the phonological processing pathway, and indicates that young VLBW adolescents may compensate for a dysfunctional phonological processing system by recruiting the left IFG during phonological processing, while during orthographic and semantic processing a decrease in activity was observed for VLBW. The activation and deactivation observed in correlation with altered cognitive performance unrelated to group was located in the right hemisphere, except for the observed higher accuracy in semantic processing correlated to left angular gyrus activation that seemed specific for VLBW. The VLBW group showed impaired performance on the WISC Block Design test, but not during the WISC Vocabulary or the fMRI language tests measuring neural patterns related to decoding and language comprehension. Therefore, we suggest that decreased WISC Block Design performance is involved in the development of changed functional activation patterns, however is not directly affecting reading ability performance. In conclusion, we have observed an aberrant neural activation and deactivation pattern for reading ability components, that appears to be effective in compensating for potential underperformance on decoding and language comprehension tasks, and is dissimilar from cognitive impairment effects.

## Supporting information

S1 Table Participant data, cognitive measures from the WISC tests, and fMRI performance data. g = grams, n = number, SD = standard deviation, ms = milliseconds, mm3 = cubic millimeters.
(XLS)
